# Correlating Molecular and Textural Properties of Raw Soy‐Based and Beef Burgers Using TD‐NMR and TPA

**DOI:** 10.1111/1750-3841.70839

**Published:** 2026-01-09

**Authors:** Moshe Hai Azachi, Zeev Wiesman

**Affiliations:** ^1^ Phyto‐Lipid Biotech Lab (PLBL) Department of Biotechnology Engineering, Faculty, of Engineering Sciences Ben Gurion University of the Negev Beer Sheva Israel

**Keywords:** Angus beef, food quality control, soy‐based burger, structural analysis, TD‐NMR, TPA, texture optimization, water‐holding capacity (WHC), water mobility

## Abstract

The accelerating demand for plant‐based meat alternatives necessitates advanced analytical approaches capable of characterizing and optimizing texture—a key determinant of consumer acceptance. This study develops an integrated framework combining time‐domain nuclear magnetic resonance (TD‐NMR) and texture profile analysis (TPA) to elucidate the molecular, structural, and mechanical differences between soy‐based plant‐based meat analogue (PBMA) burgers and conventional Black Angus beef burgers. Gravimetric and centrifugal assays showed that Angus beef possessed higher total water content and a greater proportion of free and loosely bound water than Soy‐PBMA, reflecting stronger water–protein associations in the myofibrillar muscle matrix. TD‐NMR relaxation and self‐diffusion analyses further demonstrated longer *T_1_*/*T_2_* components and higher diffusion coefficients in beef, indicating enhanced water and lipid mobility, improved phase dispersion, and a more cohesive intracellular structure. These molecular features were consistent with TPA results, in which beef exhibited significantly higher hardness, cohesiveness, springiness, and chewiness, reflecting its dense fibrous architecture. Pearson correlation analysis revealed strong associations between NMR parameters and TPA metrics, establishing a mechanistic linkage between water mobility, matrix confinement, and macroscopic texture behavior. Complementary cryo‐scanning electron microscopy (Cryo‐SEM) imaging visualized these structural contrasts, showing tightly aligned muscle fibers and well‐integrated fat globules in beef versus a porous, heterogeneous protein–starch–lipid network in Soy‐PBMA. Together, these findings demonstrate that the integrated TD‐NMR–TPA approach provides a powerful, non‐destructive tool for connecting molecular‐scale water dynamics with functional textural properties. This framework offers a predictive foundation for improving formulation, processing, and quality control in next‐generation plant‐based burgers.

## 1 Introduction

The global demand for plant‐based meat alternatives has increased sharply in recent years, driven by growing consumer awareness of sustainability, health, and animal welfare concerns. Among these alternatives, soy‐based burgers have become a leading product category due to their high protein content, favorable amino acid profile, and functional versatility (McClements 2020; Sha and Xiong 2020). Despite their widespread adoption, replicating the texture and mouthfeel of conventional meat remains one of the most significant formulation challenges in plant‐based foods (Dekkers et al. 2016, Dekkers et al. 2018; Konrad et al. 2024). Because texture strongly influences consumer expectations of quality, juiciness, and overall palatability, it plays a central role in determining product acceptance.

WHC is a key physicochemical property governing texture, cooking yield, and juiciness. WHC refers to the ability of a food matrix to retain water during processing, storage, and heating (Bertram et al. 2004a; Ofstad et al. 1995). In conventional meats such as Angus beef, high WHC is attributed to the abundance of myofibrillar proteins—particularly myosin—that effectively bind water, as well as to the structural integrity of muscle fibers and capillary networks that help confine intracellular moisture. Reported WHC values for raw Angus beef burgers typically range from 75–85%, with drip losses below 2–3% during cold storage (Ahmad et al. 2022; Konrad et al. 2024).

In contrast, soy‐based PBMA burgers generally exhibit lower and more variable WHC, depending on formulation. Although soy protein isolates (SPI) and concentrates can bind water, they are typically less effective than myofibrillar proteins. Textured soy protein (TSP) contributes to water retention through its sponge‐like structure but tends to release moisture under mechanical or thermal stress. To improve WHC, formulators often incorporate hydrocolloids (e.g., methylcellulose, carrageenan) and emulsifiers. Depending on protein concentration and processing conditions, the WHC of raw soy‐based PBMA burgers usually ranges from 60–75%, with drip losses up to 5% in the absence of hydrocolloid modification (Konrad et al. 2024).

SEM is commonly employed to visualize the microstructure of meat and plant‐based analogues. SEM provides high‐resolution images of protein networks, fat globules, and water‐retaining micro domains, thereby offering insights into structural features underlying textural behavior (Abdullah et al. 2024; Gordon and Barbut 1990).

TPA is widely used to quantify mechanical textural attributes, including hardness, cohesiveness, springiness, and chewiness (McClements et al. 2021). This two‐cycle compression test simulates mastication and captures bulk mechanical responses (Trinh and Glasgow 2012). However, while TPA provides valuable macro‐scale texture information, it does not directly probe underlying molecular interactions—such as water–protein binding or fat‐phase mobility—that critically influence the performance of plant‐based matrices.

TD‐NMR complements TPA by offering non‐destructive insight into internal matrix dynamics. Through measurements of proton relaxation times (*T_1_* and *T_2_*), TD‐NMR can differentiate among tightly bound, entrapped, and free water populations, as well as assess fat mobility, crystallinity, and phase transitions (Dekkers et al. 2018; Schreuders et al. 2021). Its sensitivity to hydration, protein structuring, and lipid behavior makes TD‐NMR particularly powerful for studying structure–function relationships in complex food systems (Van Duynhoven et al. 2010; Wiesman et al. 2025).

Despite the strengths of each technique, their combined application for optimizing the texture of plant‐based meat remains limited. Integrating TD‐NMR with TPA enables direct mechanistic‐to‐functional linkage: molecular‐scale mobility data from NMR can be correlated with macro‐scale mechanical outcomes, providing a comprehensive understanding of texture formation. This synergy supports rational formulation design, process optimization, and data‐driven quality control in the rapidly evolving alternative protein sector (Callaghan 2008; Galvosas et al. 2019).

This study addresses this gap by employing TD‐NMR and TPA in tandem to characterize the texture and water dynamics of soy‐based burger patties. By systematically examining relaxation behavior, water mobility, and mechanical properties, we identify quantitative correlations between molecular mobility and macroscopic texture. The overarching objective is to establish a predictive framework for texture optimization and process control in soy‐based PBMAs, thereby advancing both scientific understanding and product quality.

## 2 Materials and Methods

### 2.1 Materials

This study analyzed two commercial burger types: animal‐based Black Angus beef burgers and plant‐based Tivall Vegan Soybean PBMA burgers. The Angus burgers were sourced from Loup River Beef (Nebraska, USA), and the Soy‐PBMA burgers were produced by Osem (Israel). Both products were obtained from local retail suppliers.

The Black Angus burgers consisted primarily of beef, water, proteins, and fats. Some formulations also included salt, wheat fiber, black pepper, and preservative sodium metabisulfite (E‐223). Standard production involved trimming, grinding, and mixing of the ingredients.

The Tivall Soy‐PBMA burgers were composed of textured vegetable protein (water, soy protein concentrate, and wheat starch containing gluten), vegetable oils (rapeseed and sunflower), water, corn starch, methyl cellulose (thickener), salt, natural flavorings, maltodextrin, barley malt extract, yeast extract, spices (e.g., black pepper, cumin, and rosemary), baking powder (diphosphates and sodium carbonates), and rice flour.

#### 2.1.1 Sample Preparation

All samples were purchased frozen and stored at −20°C. Prior to analysis, they were thawed at room temperature (24°C) to simulate typical consumer handling. Thawed samples were then equilibrated at 40°C for 30 min before measurements to ensure consistent thermal conditions for TD‐NMR and TPA analyses.

### 2.2 Low‐Field NMR Analysis

Instrument Setup: Proton (^1^H) LF‐NMR measurements were conducted on a Maran bench‐top pulsed NMR analyzer (Resonance Instruments, Witney, UK), operating at 23.4 MHz with an 18 mm probe. All samples were stabilized and equilibrated at 40°C before measurements.

Relaxation Measurements: T_2_ (spin–spin) relaxation times were acquired using the Carr–Purcell–Meiboom–Gill (CPMG) pulse sequence (Carr and Purcell 1954; Meiboom and Gill 1958). T_1_ (spin–lattice) relaxation times were obtained via an inversion recovery pulse sequence. 2D *T_1_*–*T_2_* cross‐correlation experiments used an Inversion Recovery–CPMG sequence (Resende et al. 2020). Signal processing employed a primal‐dual convex objectives (PDCO) inverse Laplace transform algorithm with a regularization factor of α = 0.5 (Berman et al. 2013; Campisi‐Pinto et al. 2018).

Self‐diffusion measurements: elf‐diffusion coefficient (*D*) measurements were performed using a 20 MHz minispec LF‐NMR analyzer (Bruker Analytik GmbH, Berlin, Germany) with a 10 mm probe and temperature‐controlled chamber. The method followed Aghelnejad et al. 2022 and Stejskal and Tanner 1965. Samples were equilibrated at 40°C before measurement. Technique: pulsed‐field gradient spin echo (PFGSE). Parameters: 16 scans, τ = 7.5 ms, recycle delay = 6 s Gradient settings: Δ = 7.5 ms, δ = 0.5 ms, delay = 1 ms, G = 1.6 T/m. Each D value reflects the mean of 10 replicate scans per sample.

### 2.3 TPA

TPA was performed using a TA.XT Plus Texture Analyzer (Stable Micro Systems, Surrey, UK) with a 50 kg load cell and a 75 mm flat cylindrical probe, following Trinh and Glasgow 2012, with minor optimizations.

Each sample underwent a double compression cycle (two‐bite test) simulating mastication. The following mechanical parameters were extracted from the force–time curves: Hardness: maximum force in the first compression. Springiness: Distance recovered between compressions. Cohesiveness: ratio of the second to the first area under the curve. Chewiness: hardness × cohesiveness × springiness. Resilience: ratio of energy recovered during decompression to that input during compression.

Test conditions: Pre‐test speed: 1.0 mm/s. Test speed: 2.0 mm/s. Post‐test speed: 1.0 mm/s. Compression depth: 50% of sample height. Time between compressions: 5 s. Trigger force: 10 g.

All measurements were performed in triplicate (*n* = 15) for each sample. Data are reported as mean ± standard deviation (SD).

### 2.4 Cryo‐SEM

Samples were cryo‐immobilized between two aluminum discs (3 mm diameter, 200 µm thick each) using a high‐pressure freezer (EM ICE, Leica). Sample preparation and imaging followed this workflow: Transferred under liquid nitrogen to the EM VCM station, then cryogenically transferred via EM VCT500 to the EM ACE900 freeze‐fracture device → Fractured at −140°C with a cooled knife to expose internal morphology → Etched at −100°C for 5 min to sublimate surface water → Coated with a 3 nm platinum layer. Imaging was performed using a Gemini SEM (Zeiss) with an in‐lens secondary electron detector at −120°C operating temperature (Sharma and Bhardwaj 2019).

All imaging was conducted at the Ilse Katz Institute for Nanoscale Science and Technology, Ben‐Gurion University of the Negev, Beer Sheva, Israel.

### 2.5 Water Content Analysis

Gravimetric water content—raw burger samples were weighed, dried at 100°C for 24 h, and reweighed. Water content was calculated gravimetrically following the method of (Kolar 1992).

Centrifugal water release—additional samples were weighed, placed in metal‐filtered centrifuge tubes, and centrifuged at 1500 g for 20 min, a protocol selected to minimize structural deformation. Released water was quantified as described by (Barbut 2024).

#### 2.5.1 Determination of Bound and Immobilized Water Fractions

The fractions of free, immobilized, and bound water were quantified by integrating gravimetric measurements with centrifugal separation and TD‐NMR relaxation/diffusion analyses.

Moderate centrifugation at 1500 × g for 20 min removed predominantly free and loosely associated water, as evidenced by the decrease in total sample water mass following centrifugation. The water remaining within the sample after centrifugation was operationally defined as the immobilized water fraction, comprising capillary‐entrapped water and water interacting strongly with proteins, polysaccharides, or other structural components.

Immediately after centrifugation, samples were sealed to prevent evaporative loss and analyzed by TD‐NMR. The post‐centrifugation diffusion coefficient (*D_2_*) reflects the mobility of water confined within the matrix, which is substantially lower than the diffusion coefficient of untreated samples (*D_1_*). Thus, *D_2_* characterizes matrix‐associated water mobility rather than bulk or free‐water diffusion.

The proportion of immobilized water was calculated from the mass of water retained after centrifugation relative to the total water content obtained by gravimetry. This estimate was cross‐validated using TD‐NMR relaxation data: the amplitude of short‐*T_2_* components (<10 ms) in CPMG decay curves was used as a qualitative indicator of tightly associated or entrapped water populations, consistent with prior literature (Bertram et al. 2004b; Van Duynhoven et al. 2010).

Ratios of *D_1_* to total water content and *D_2_* to immobilized water content were calculated using immobilized‐water percentages reported in Table 1, providing an internal check on water‐mobility scaling relative to the gravimetrically determined fractions.

**TABLE 1:** WHC of raw Angus‐beef and Soy‐PBMA burgers. Dry matter and total water content (%) were determined by oven‐drying at 100°C for 24 h. Free and loosely bound water (%) was quantified by moderate centrifugation at 1500 × g for 20 min. Immobilized water (%) was calculated as the difference between total water content (%) and centrifuge‐released water (%). Data are presented as mean ± SD.

Sample	Dry weight %*	Total water %	Centrifuge released water %	Immobilized water %**
Angus‐beef burger	31.1 ± 2.5	68.9 ± 2.5	11.7 ± 2.4	56.35 ± 1.1
Soy‐PBMA burger	39.2 ± 1.6	60.8 ± 1.6	8.3 ± 0.8	52.38 ± 2.2

*Notes*: *Dry weight—sample weight after oven heating (% from initial weight).

**Immobilized water is calculated by % total water content—% centrifuge released water.

In summary, *D_2_* and short‐*T_2_* relaxation components serve as proxies for immobilized/bound water, whereas *D_1_* and long‐*T_2_* components represent more mobile water fractions. Each burger type was characterized by using six independent samples, with ten replicate diffusion measurements performed per sample.

### 2.6 Statistical Analysis

**FIGURE 1:**
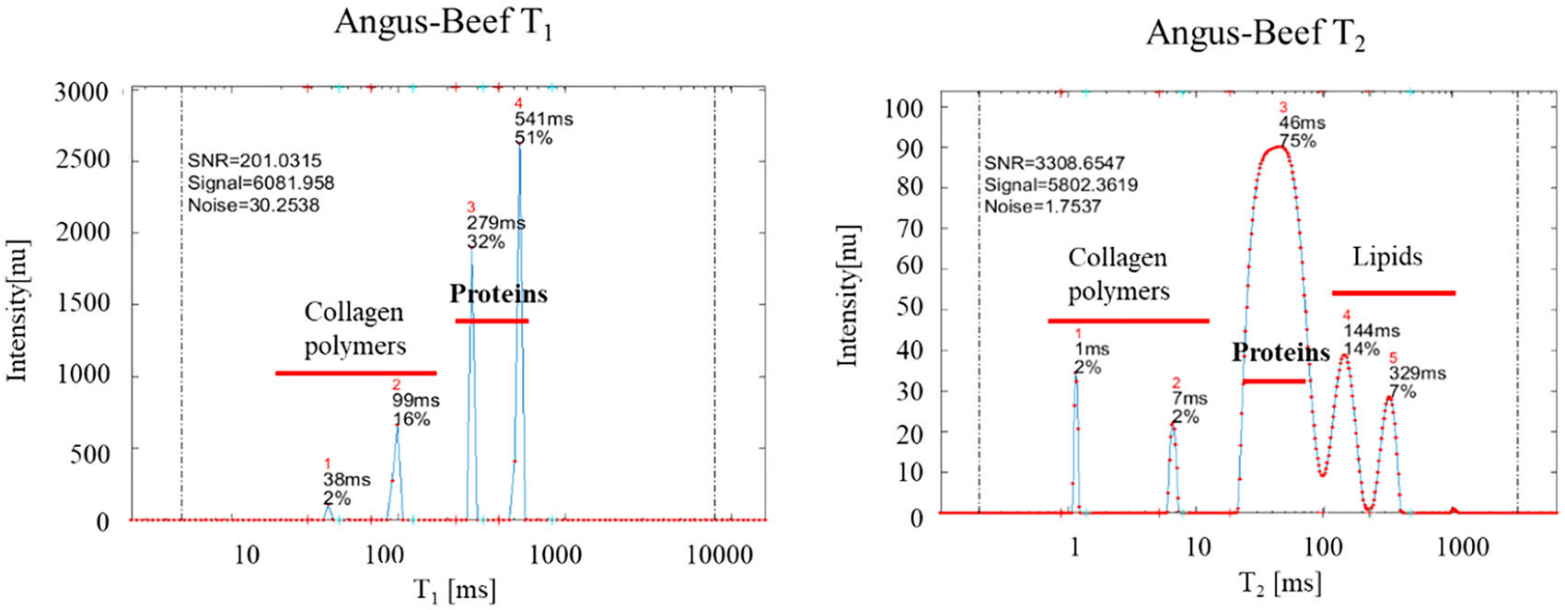
Representative TD‐NMR 1D *T_1_* recovery (left) and *T_2_* decay (right) spectra of raw Angus‐beef burger. Shown are typical longitudinal (*T_1_*) recovery and transverse (*T_2_*) decay profiles, selected from a dataset of 64 replicate measurements. These profiles illustrate the reproducible relaxation behavior of water and fat components in the raw Angus‐beef matrix.

**FIGURE 2:**
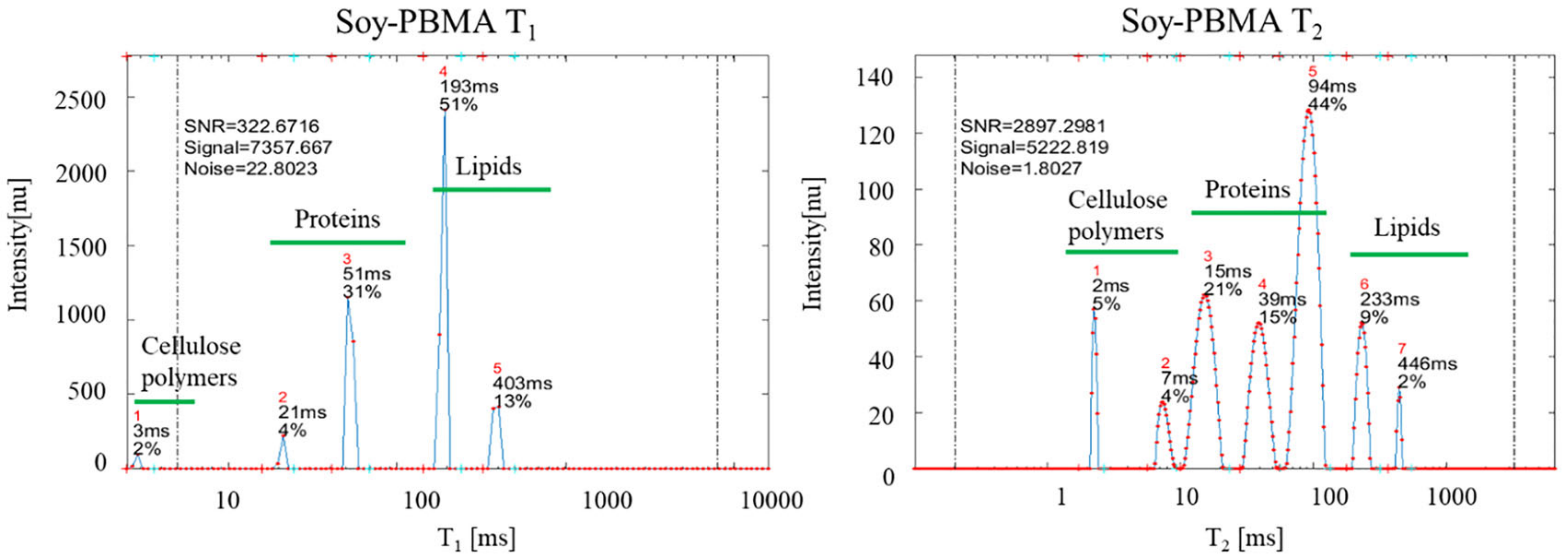
Representative TD‐NMR 1D *T_1_* recovery (left) and *T_2_* decay (right) spectra of raw Soy‐based PBMA (Soy‐PBMA) burger Displayed are representative longitudinal (*T_1_*) recovery and transverse (*T_2_*) decay curves, selected from a dataset of 64 replicate measurements. These profiles illustrate the characteristic relaxation behavior of water and fat compartments within the Soy‐PBMA matrix.

**FIGURE 3:**
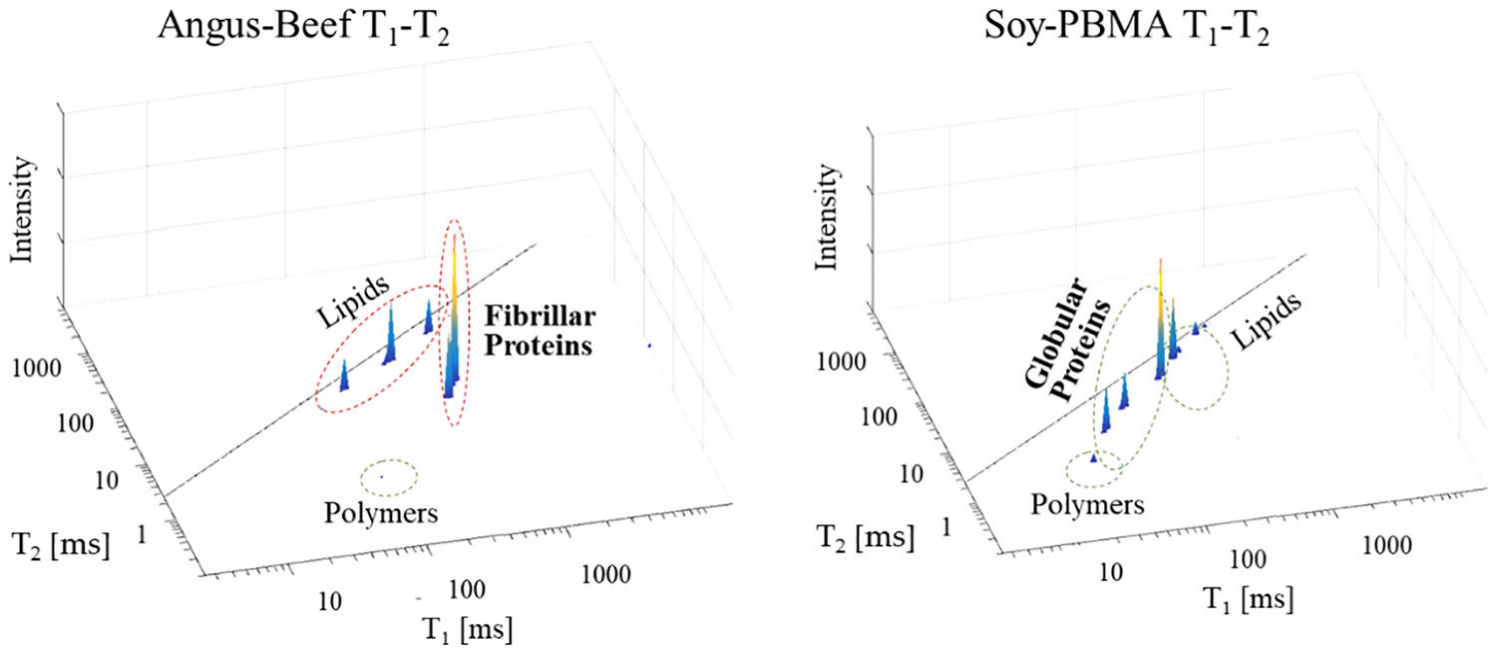
Representative TD‐NMR 2D *T_1_*–*T_2_* correlation spectra of raw Angus‐beef (left) and Soy‐based PBMA (Soy‐PBMA) burgers (right). These representative 2D *T_1_*–*T_2_* spectra were selected from a dataset of 64 replicate measurements. The main cluster of peaks corresponding to myofibrillar proteins in Angus beef appears at *T_1_* relaxation times above 500 ms, whereas the primary peaks assigned to globular soy proteins in Soy‐PBMA are observed at *T_1_* times of approximately 100 ms or lower. Additional distinct groups corresponding to lipids and polymeric components are also indicated.

T_1_ and T_2_ relaxation decay/recovery curves (Figures 1, 2) and 2D *T_1_*–*T_2_* correlation maps (Figure 3) were generated from 15 replicates per burger type. Water content and centrifugal water‐release measurements (Table 1) were based on six replicates per condition and are reported as mean ± SD.

**FIGURE 4:**
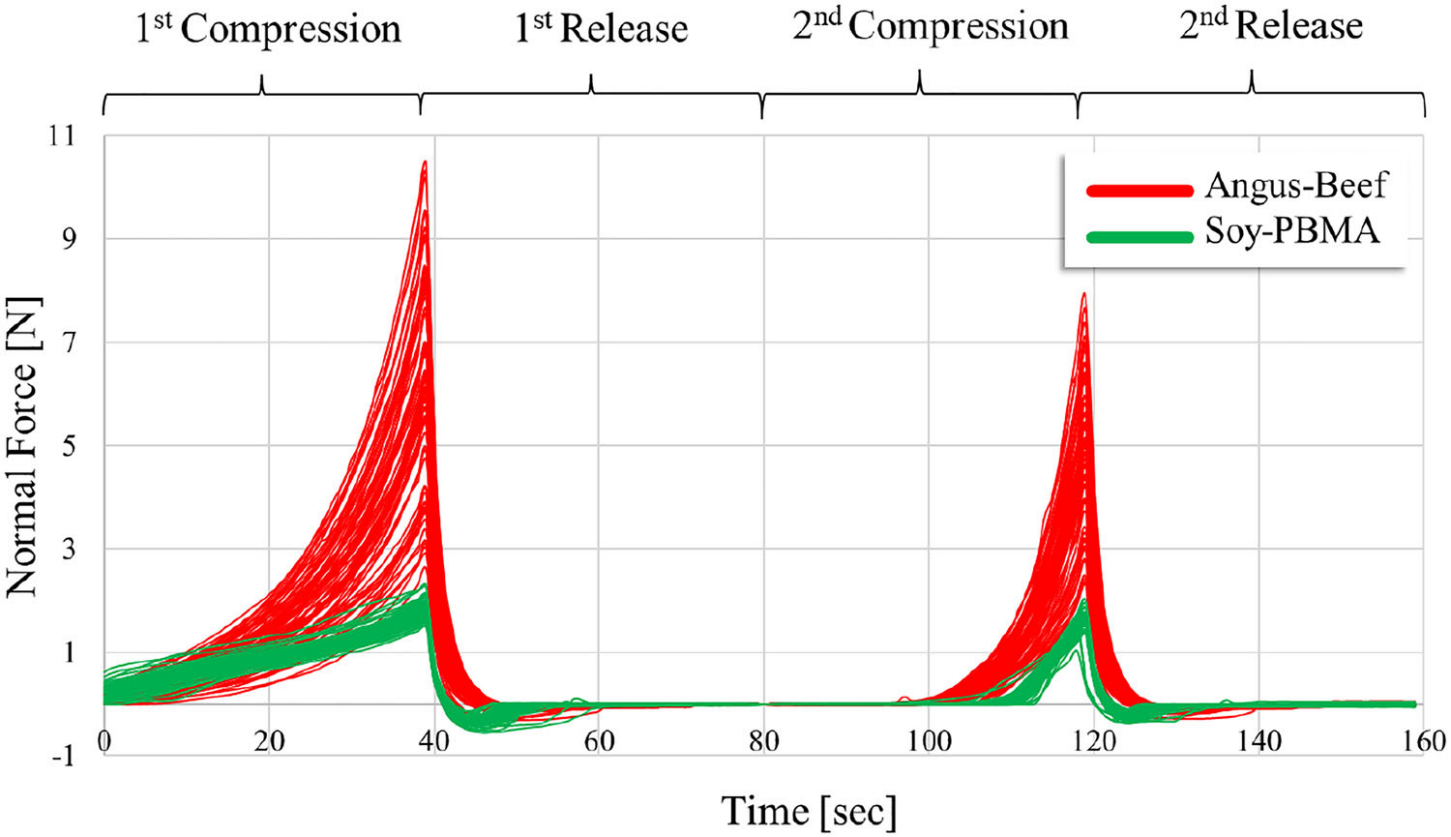
Comparison of TPA “two‐bite” deformation profiles of Angus‐beef and Soy‐PBMA burgers. In both compression cycles (“bites”), Angus‐beef burgers exhibited significantly higher forces than Soy‐PBMA burgers, reflecting distinct differences in their textural properties.

*D* for raw, untreated samples (Table 2) were determined from 30 measurements per burger type. TPA results (Figure 4, Table 3) were obtained from a large internal database comprising more than 100 independent replications.

**TABLE 2:** *D* of raw Angus‐beef and Soy‐PBMA burgers. Coefficient *D_1_* was measured in fresh (untreated) burger samples, while coefficient *D_2_* was measured after moderate centrifugation at 1500 × g to remove free and loosely bound water. Ratios *D_1_* / total water content and *D_2_* / immobilized water content were calculated using immobilized water percentages reported in Table 1. Data are presented as mean ± SD.

Sample	Coefficient *D1* raw burger (10^−9^m^2^/s)	Ratio of *D_1_* / total water	Coefficient *D2* follows centrifuge (10^−9^m^2^/s)	Ratio of Coeff. *D2* / immobilized water
Angus‐beef burger	1.46 ± 0.12	0.021	1.39 ± 0.045	0.024
Soy‐PBMA burger	1.37 ± 0.05	0.022	1.34 ± 0.056	0.025

*Note*: Data are presented as mean ± SD.

**TABLE 3:** Comparison of TPA “two‐bite” parameters for Angus‐beef and Soy‐PBMA burgers. Average values of TPA parameters—Including hardness, cohesiveness, springiness, and chewiness—Are shown for Angus‐beef and Soy‐PBMA burgers. All values are expressed in units of normal force (*N*). Data are presented as mean ± SD.

Sample	Hardness	Cohesiveness	Springiness	Chewiness
Angus‐beef burger	6.26 ± 1.88	0.42 ± 0.04	0.43 ± 0.09	1.18 ± 0.54
Soy‐PBMA burger	1.85 ± 0.27	0.22 ± 0.04	0.26 ± 0.06	0.09 ± 0.04

*Note*: All values are expressed in units of normal force (N). Data are presented as mean ± SD.

Correlations between TPA parameters and NMR‐derived diffusion coefficients (Table 4) were evaluated using Pearson's correlation coefficient (*r*). Associated P‐values were used to assess statistical significance and the probability of false‐positive associations.

**TABLE 4:** Statistical correlations between TD‐NMR *D* and TPA parameters. Correlation coefficients quantifying the relationship between diffusion coefficient *D* values obtained from TD‐NMR measurements and TPA parameters are presented.

TPA parameter	NMR parameter	Pearson correlation *r* *	*P*‐value**
Hardness	Coefficient *D*	0.75	8.29·10^−7^
Cohesiveness	Coefficient *D*	0.76	2.18·10^−6^
Springiness	Coefficient *D*	0.73	7.99·10^−6^
Chewiness	Coefficient *D*	0.75	3.04·10^−6^

* Pearson *r* values above 0.7 indicate a strong correlation.

** *P*‐values represent the probability that the observed correlation occurred by chance.

## 3 Results and Discussion

Water is a critical determinant of texture, juiciness, and overall sensory quality in both animal‐ and plant‐based burgers. Beyond serving as a filler, water contributes to tenderness, mouth feel, cooking behavior, and structural integrity, directly influencing consumer acceptance. In this study, we characterized water status and its impact on texture in Angus beef and Soy‐PBMA burgers using thermogravimetry, TD‐NMR, TPA, and Cryo‐SEM imaging.

### 3.1 WHC of Raw Burgers

Thermogravimetric analysis (100°C, 24 h) showed that raw Angus beef burgers contained 68.9% water and 31.1% dry matter, whereas Soy‐PBMA burgers contained 60.8% water and 39.2% dry matter (Table 1). Under moderate centrifugation (1500 g), Angus burgers released 11.7% of their total water as free or loosely bound water, compared with 8.3% released from the Soy‐PBMA samples—representing the easily mobilizable water fraction.

Strongly bound water in protein‐rich matrices typically accounts for 4–8% of total water (Cooke and Wien 1971; Khan et al. 2016). Using this range, the remaining water can be categorized as immobilized water, encompassing both strongly associated water and more loosely entrapped or emulsified water. Based on this partitioning, Angus beef contains approximately 56.3% immobilized water, whereas Soy‐PBMA contains about 52.3%. This immobilized fraction corresponds to water associated with the structural protein network that is not released under mild mechanical stress.

In Angus beef, most water resides intracellularly and is stabilized through interactions with myofibrillar proteins such as actin and myosin. These strong protein–water interactions contribute to the characteristic fibrous texture and juiciness of muscle tissue (Bertram et al. 2004b).

By contrast, the Soy‐PBMA matrix retains a substantial portion of its water extracellularly within a plant‐protein gel network. Water–matrix interactions in this system are generally weaker and more heterogeneous, making it more susceptible to drip loss during processing or cooking. Functional ingredients such as methylcellulose improve water retention by increasing viscosity and gel strength, but they do not replicate the binding strength of native muscle proteins.

Overall, the WHC differences underscore fundamental microstructural distinctions between muscle‐based and plant‐based matrices: beef relies on an organized myofibrillar architecture for stable water entrapment, whereas Soy‐PBMA depends on a reconstructed plant‐protein gel network with inherently lower water‐binding capacity.

### 3.2 TD‐NMR Relaxation Microstructural Characterization

TD‐NMR provided detailed insight into water distribution and mobility at the molecular level (Bertram, Purslow, et al. 2002; Dekkers et al. 2018; Van Duynhoven et al. 2010).

#### 3.2.1 Angus Beef

T_1_ peaks at 541 ms and 279 ms, attributed to myofibrillar water, contributed 51% and 32% of the signal, respectively (Figure 1). Short‐T_1_ components were associated with collagen. *T_2_* relaxation exhibited a dominant peak at 46 ms (75% of signal), representing immobilized intracellular water, while longer peaks at 144 ms and 329 ms corresponded to lipids.

#### 3.2.2 Soy‐PBMA

*T_1_* peaks at 193 ms and 51 ms, corresponding to soy proteins, accounted for 51% and 31% of the signal (Figure 2). Short‐T_1_ components (3 ms, 21 ms) were assigned to cellulose, and a longer peak at 403 ms reflected lipids. T_2_ showed three protein‐associated peaks at 94 ms (44%), 39 ms (15%), and 15 ms (21%), with lipid peaks at 233 ms and 446 ms.

#### 3.2.3 2D *T_1_*–*T_2_* Correlation Spectra

2D *T_1_*–*T_2_* maps (Figure 3) distinguished the two matrices: Angus beef exhibited *T_1_* > 500 ms and *T_2_* < 100 ms, characteristic of tightly confined intracellular water, while Soy‐PBMA showed *T_1_* < 100 ms and T_2_ between 20–200 ms, indicating more mobile extracellular water. Lipid‐associated peaks were broader in Soy‐PBMA, and short *T_1_*–*T_2_* signatures captured structural polymers (collagen in beef, cellulose in soy).

### 3.3 *T_1_*, *T_2_*, and *D*: Water Mobility and Structural Confinement

Water content strongly influences NMR relaxation. Increasing free or loosely immobilized water elevates *T_1_*, while tightly bound water shortens it. In structured matrices, water may remain immobilized, moderating these effects. *T_2_* values reflect mobility: short *T_2_* indicates bound/intracellular water; long *T_2_* indicates free water. Broadening of *T_2_* distributions reflects multiple water populations (Bertram et al. 2002; Wang et al. 2020; Wiesman et al. 2025).

*D* complements relaxation data. Higher *D* indicates mobile water; lower *D* reflects restricted diffusion. In untreated samples, *D_1_* = 1.46 × 10^−9^ m^2^/s (Angus) and 1.37 × 10^−9^ m^2^/s (Soy‐PBMA). After moderate centrifugation, *D_2_* decreased to 1.39 × 10^−9^ m^2^/s (Angus) and 1.34 × 10^−9^ m^2^/s (Soy‐PBMA), reflecting residual immobilized water mobility.

Centrifugation removes free and loosely bound water, so *D_2_* measures the remaining immobilized fraction. Lower *D_2_* indicates stronger water–matrix interactions and higher immobilization (Angus), whereas higher *D_2_* reflects weaker confinement (Soy‐PBMA). These findings align with WHC trends.

### 3.4 TPA Texture Analysis of Raw Burgers

TPA revealed pronounced textural differences (Figure 4, Table 3). Angus beef had higher hardness (6.255 *N*), cohesiveness (0.418), springiness (0.434), and chewiness (1.176) than Soy‐PBMA (1.848 N, 0.224, 0.225, 0.094). These values reflect the fibrous, elastic structure of meat versus the softer, homogeneous soy matrix. Soy‐PBMA's lower resilience and chewiness correspond to the absence of organized fibrous networks. Structural additives (texturized soy protein, hydrocolloids, emulsifiers) are often incorporated into mimic meat‐like mechanics (Mabrouki et al. 2023).

### 3.5 Correlation Between TD‐NMR and TPA Measurements

Pearson correlations showed *D* significantly correlated with hardness, cohesiveness, springiness, and chewiness (*r* = 0.73–0.77, Table 4), indicating that molecular water mobility is predictive of macroscopic texture. TD‐NMR thus provides a mechanistic link to TPA metrics, capturing water retention, matrix continuity, and viscoelastic behavior.

This approach is supported by prior studies (Althoff et al. 1996; Callaghan 2008; Dekkers et al. 2016; Galvosas et al. 2019; Schreuders et al. 2021), and advances in Rheo‐NMR provide further evidence for non‐destructive texture prediction (Xiong et al. 2024). Although expanding sample sets would enhance generalizability, this proof‐of‐concept study demonstrates feasibility in representative burger systems.

### 3.6 Morphological Analysis

**FIGURE 5:**
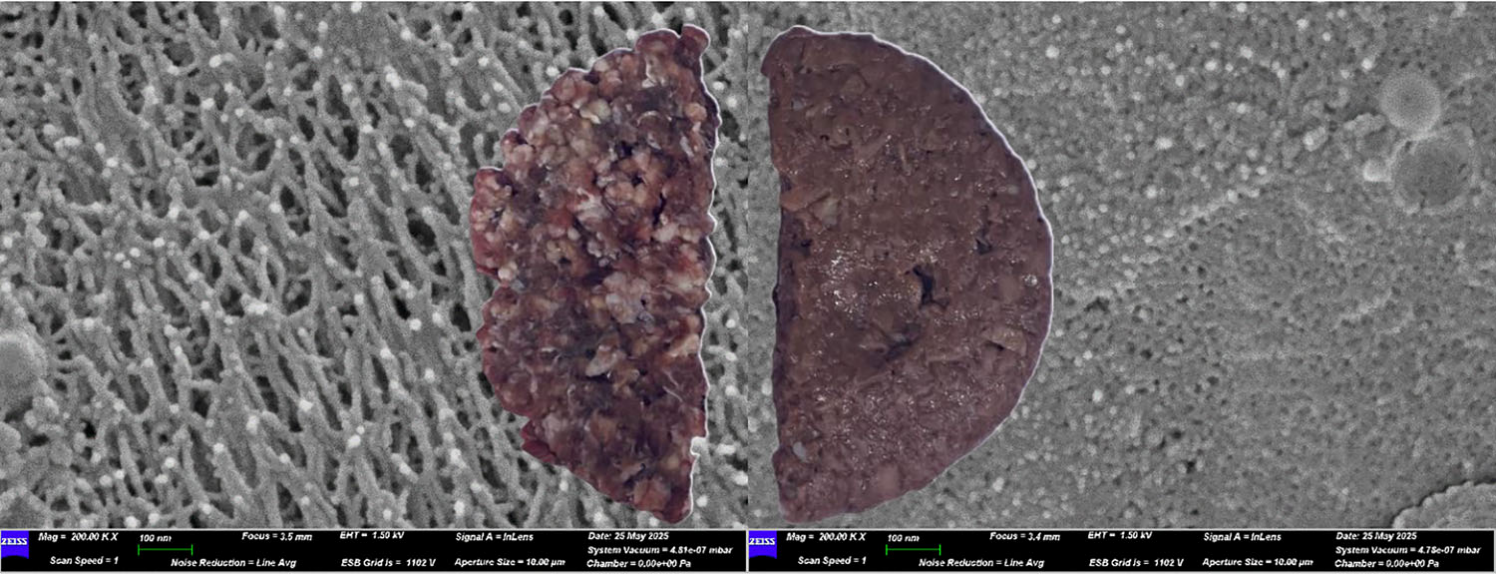
Combined visual and Cryo‐SEM microstructural comparison of Angus meat and Soy‐PBMA burgers. The left panels show the eye‐visible appearance and Cryo‐SEM micrograph (magnification 200,000×) of the Angus meat burger, characterized by an aggregated, bundle‐like structure with a wide range of spherical particle sizes and a fibrillar rod‐like microstructure. The right panels depict the Soy‐PBMA burger's visual appearance and corresponding Cryo‐SEM image, revealing a well‐dispersed pattern of emulsified spherical vesicles of varying sizes and a condensed emulsified microstructure.

Macroscopic observations revealed marked differences between the two burger types (Figure 5). Angus beef appeared red, with visible marbling, a coarse fibrous texture, and an irregular shape, whereas Soy‐PBMA was beige, uniform, and smooth, with evenly distributed fat droplets. Beef exhibited higher glossiness, while Soy‐PBMA appeared to be matte or slightly oily. These macroscopic features reflect underlying micro scale structural differences, as confirmed by Cryo‐SEM analysis.

### 3.7 Cryo‐SEM Analysis of Burger Microstructure

#### 3.7.1 Angus Beef

Cryo‐SEM imaging revealed a densely packed, fibrous microstructure characteristic of intact bovine muscle. The matrix consisted of elongated myofibrils interspersed with intramuscular fat globules and connective tissue (Figure 5). This heterogeneous yet well‐organized architecture produced small and relatively uniform pore spaces, resulting in limited inter‐fiber voids.

These microstructural features align closely with the functional properties measured by TD‐NMR and TPA. The restricted pore volume and tight filament packing correspond to the high WHC and limited water mobility observed in diffusion measurements. Literature reports indicate that much of the immobilized water in muscle resides within the inter‐ and intra‐myofibrillar compartments, where inter‐filament spacing typically falls below ∼10 µm and can approach sub‐micron dimensions depending on rigor state and ionic strength (Huff‐Lonergan and Lonergan 2005). Such confinement supports the robust cohesiveness, springiness, and tenderness measured instrumentally.

In addition, the presence of marbling contributes to overall mechanical stability by reinforcing the fibrous network and enhancing tenderness. Together, the tightly packed protein filaments, limited pore spaces, and embedded fat structures explain the strong structural integrity and texture attributes of the Angus beef burgers.

#### 3.7.2 Soy‐PBMA

Cryo‐SEM imaging of the soy‐based PBMA revealed a comparatively homogeneous, sponge‐like matrix composed of interwoven protein, starch, and lipid domains (Figure 5). The structure featured large, irregularly shaped voids dispersed throughout the matrix, in sharp contrast to the tightly packed, fibrous architecture of beef.

This elevated porosity and the discontinuous nature of the protein–fat network are consistent with the lower WHC, broader *T_2_* relaxation distributions, and reduced *D_2_* obtained from TD‐NMR analysis. The large pore volumes facilitate greater mobility of unbound or weakly bound water, leading to diminished mechanical resistance and correspondingly lower TPA metrics—including hardness, cohesiveness, and springiness.

Published characterizations of PBMAs support these observations. High‐moisture meat analogues (HMMA) commonly exhibit median pore diameters of ∼30–40 µm, depending on formulation and aeration conditions (Zink et al. 2023). Micro‐CT studies of soy‐based analogues similarly report large “air‐bubble” voids and heterogeneous pore networks that far exceed the sub‐10 µm inter‐filament spaces typical of muscle tissue (Taghian Dinani et al. 2024). These literature values align closely with the sponge‐like microstructure observed in our Cryo‐SEM images and help explain the broader *T_2_* peaks and weaker texture performance relative to beef.

Although certain additives in the Soy‐PBMA formulation appear to partially reinforce structural integrity (Sha and Xiong 2020), the inherent heterogeneity and scale of the pore network remain limiting factors for achieving meat‐like textural fidelity. While the Cryo‐SEM evaluation in this study is qualitative, future work will incorporate quantitative pore‐size distribution analysis to further strengthen structure–function correlations.

#### 3.7.3 Comparative Interpretation

Cryo‐SEM analysis highlights clear structural divergence between Angus beef and Soy‐PBMA, with direct implications for water dynamics and textural performance. Beef exhibits a dense, fibrous architecture characterized by tightly packed myofibrils and small, well‐organized pore spaces (typically ≤10 µm). This fine microstructure effectively restricts water mobility, supporting the high WHC, limited diffusion, and strong mechanical properties (hardness, cohesiveness, springiness) measured by TD‐NMR and TPA.

In contrast, Soy‐PBMA displays a more porous, sponge‐like matrix containing large, irregular voids that frequently reach tens of micrometers—consistent with values reported for plant‐based analogues in the literature. These enlarged and heterogeneous pores allow freer movement of weakly bound or unbound water, contributing to broader *T_2_* relaxation peaks, lower *D*, and diminished textural attributes.

Integrating Cryo‐SEM, TD‐NMR, and TPA findings establishes a clear mechanistic framework linking pore size and connectivity to water retention and mechanical behavior across the two burger types. The pronounced pore‐size disparity between muscle and plant‐based matrices underscores a key challenge in replicating meat‐like structural fidelity. Advanced structuring strategies—such as high‐moisture extrusion, controlled protein crosslinking, or additive‐enabled alignment techniques (e.g., shear‐cell processing, 3D printing)—may help future plant‐based formulations more closely approximate the fine pore architecture and functional performance of real meat.

## 4 Conclusion

This study demonstrates that water dynamics, microstructure, and mechanical properties are tightly linked in animal‐ and plant‐based burgers. TD‐NMR revealed distinct water mobility profiles—restricted intracellular water in Angus beef versus loosely bound extracellular water in Soy‐PBMA—correlating with differences in texture measured by TPA. Cryo‐SEM confirmed these microstructural contrasts, providing a mechanistic basis for water retention and textural performance. The combined TD‐NMR–TPA approach offers a rapid, non‐destructive framework to predict and optimize plant‐based burger texture. Future work integrating sensory evaluation and quantitative microstructure analysis will be essential to fully translate molecular insights into consumer‐relevant quality. In addition, expanding the sample diversity—across protein sources, formulations, fat systems, and processing conditions—will be critical for strengthening model generalizability and validating the robustness of TD‐NMR–based texture prediction across the broader category of meat and meat‐analog products.

## Author Contributions

**Moshe Hai Azachi**: investigation, methodology, validation, visualization. **Zeev Wiesman**: conceptualization, writing – original draft, methodology, validation, writing – review and editing, supervision.

## Conflicts of Interest

The authors declare no conflicts of interest.

## Data Availability

No data was used for the research described in the article.
